# Revision of the genus *Cephalobyrrhus* of Japan and Taiwan (Coleoptera, Limnichidae)

**DOI:** 10.3897/zookeys.817.31530

**Published:** 2019-01-15

**Authors:** Hiroyuki Yoshitomi

**Affiliations:** 1 Entomological Laboratory, Faculty of Agriculture, Ehime University, Tarumi 3-5-7, Matsuyama, 790-8566 Japan Ehime University Matsuyama Japan

**Keywords:** Byrrhoidea, Cephalobyrrhinae, key, new species, taxonomy

## Abstract

Japanese and Taiwanese species of the genus *Cephalobyrrhus* are revised. A new species, *C.amami***sp. n.**, is described from Amami-Ôshima, the Ryukyus, Japan. This species is clearly distinguished from the other known species by the short and pointed median lobe and long phallobase. Two previously known species from Japan and Taiwan, *C.latus* and *C.japonicus*, are redescribed.

## Introduction

The family Limnichidae Erichson, 1846, contains four subfamilies, and is represented by approximately 400 species in 37 genera (Spangler et al. 2001; Hernando and Ribera 2005). Of these, Cephalobyrrhinae Champion, 1925 is a small subfamily consisting of four genera: *Throscinus* LeConte, 1874 (seven species) from New World; *Cephalobyrrhus* Pic, 1923 (15 species) and *Parathroscinus* Wooldridge, 1984 (five species) mainly from the Oriental Region; and *Erichia* Reitter, 1895 (= *Jaechobyrrhinus* Pütz, 1991) (one extant species) from Turkey (Jäch and Pütz 2001; Hernando and Ribera 2005; Yu et al. 2018). The most speciose genus, *Cephalobyrrhus*, was revised by Pütz (1998), and is distributed in Japan, China, India, and Nepal (Pütz 1998). However, numerous undescribed species still remain. One previously known species is recorded from each, Japan and Taiwan, but two species were revised based on limited specimens (Pütz 1998) and further study was needed.

In this paper, the Japanese and Taiwanese species of the genus are reviewed, and the description of a new species is provided.

## Materials and methods

The materials examined in this paper are deposited in the following institutions:

**EUMJ** Ehime University Museum, Matsuyama, Japan

**TARI** Taiwan Agricultural Research Institute, Taichung, Taiwan

**SEHU** Systematic Entomology, Hokkaido University, Sapporo, Japan

**NMW** Naturhistorisches Museum, Vienna

General observations and dissections were made under a Leica MZ95 stereomicroscope. Microstructures of the dissected parts in pure glycerin were studied under an Olympus BH-2 compound microscope. After observation, the dissected parts were mounted on the same card with the specimen. Photographs were taken under the Leica MZ95 and combined in Helicon Focus ver. 4.70.5 Pro (Helicon Soft Limited).

Morphological terminology follows Hernando and Ribera (2005). Morphological abbreviations used in the measurements are as follows:

**EL** length of elytra in suture;

**EW** maximum width of elytra;

**PL** length of pronotum in median line;

**PW** maximum width of pronotum;

**TL** total length (PL+EL).

The average is given in parentheses after the range.

## Taxonomy

### Cephalobyrrhus

Taxon classificationAnimaliaColeopteraLimnichidae

Pic, 1923

Cephalobyrrhus : Pic 1923: 4. Champion 1925: 174; Wooldridge 1977: 29; 1984: 121; Pütz 1998: 342.

#### Type species.

*Cephalobyrrhuslatus* Pic, 1923: 4

#### Diagnosis.

Body medium size in the family, ca 2.2–4.5 mm, oblong, weakly convex dorsally, closely covered with short setae in dorsal surface. Eyes large; distance between their inner margins approximately equal to the diameter of one eye. Mandibles (Fig. 2B) slender, with pointed tridentate apex. Maxillae (Fig. 2C) rounded in apical part of galea and lacinia, with 4-segmented palpi. Labial palpi (Fig. 2D) 3-segmented, with rhomboid terminal palpomere. Pronotum bisinuate in posterior margin, gently tapering anteriorly. Hind wings (Fig. 2A) fully developed, 2.5 times as long as wide; AA_3+4_ present; MP_3_ and MP_4_ not connected with AA_3+4_. Elytra elongate, bisinuate in basal margin, with obvious irregular zigzag markings consisting of adpressed long silver setae. Metacoxae transverse. Legs relatively long; hind tibiae exteriorly smooth; tarsal formula 5-5-5. Sexual dimorphism indistinct.

**Figure 1.**
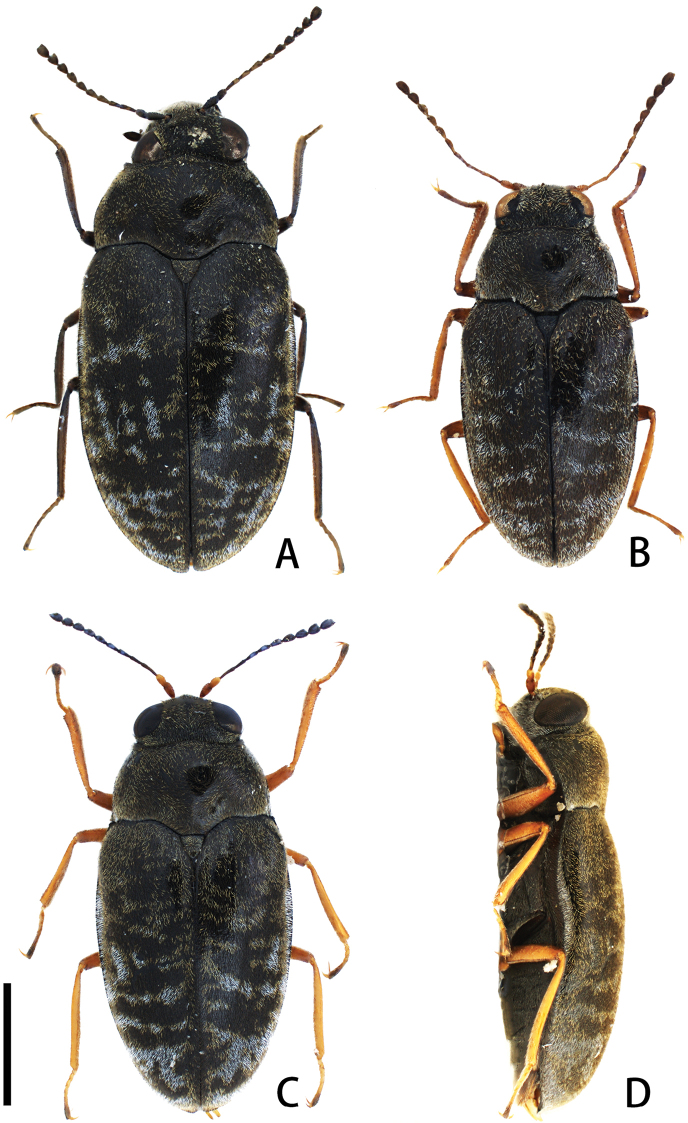
Dorsal (**A–C**) and lateral (**D**) habitus of *Cephalobyrrhus* spp. from Japan and Taiwan. **A***Cephalobyrrhuslatus* Pic, 1923 **B***C.japonicus* Champion, 1925 **C, D***C.amami* sp. n. (holotype). Scale bar: 1.0 mm.

**Figure 2.**
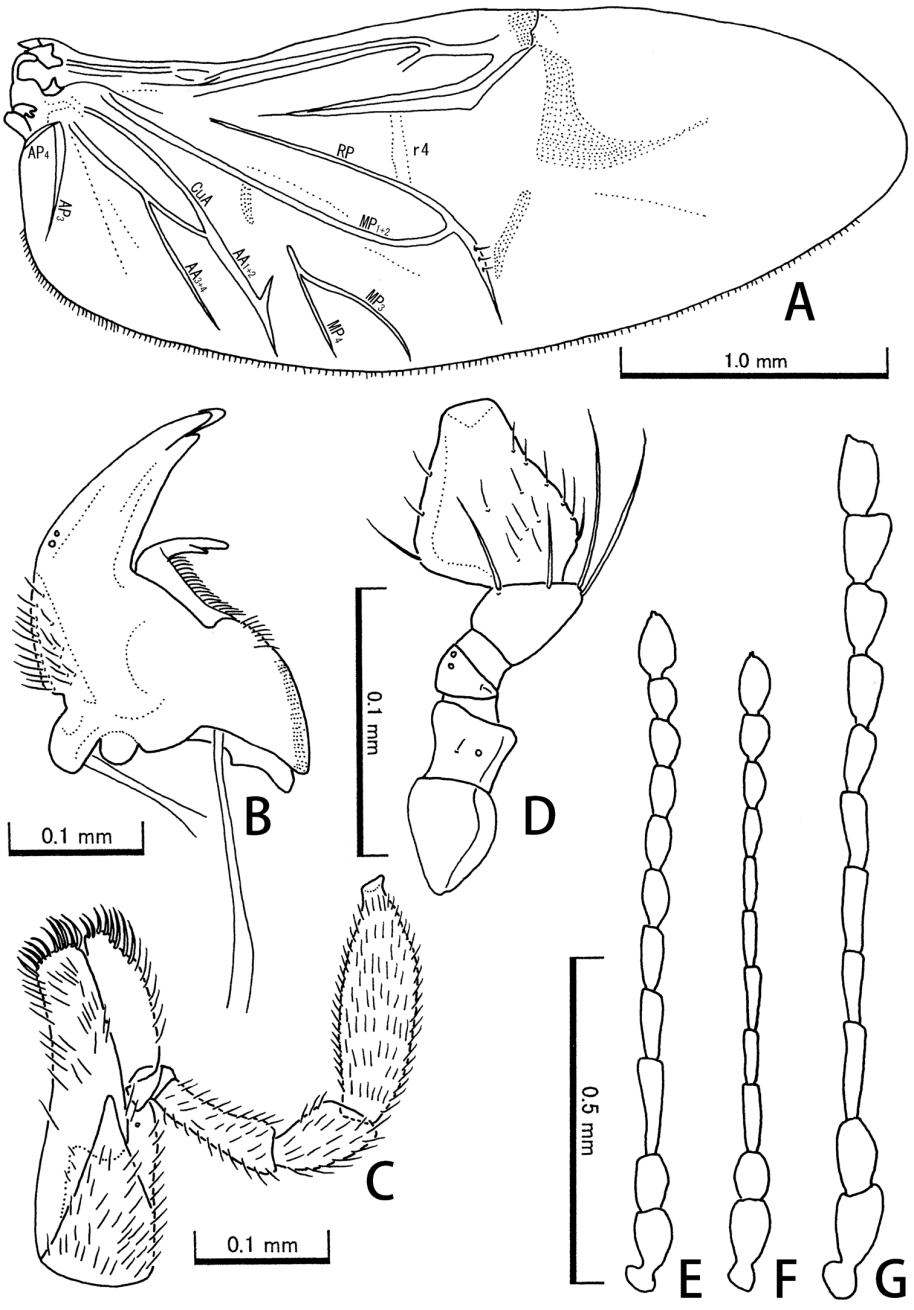
Hind wing (**A**), mandible (**B**), maxilla (**C**), labial palpus (**D**), and male antennae (**E–G**) of *Cephalobyrrhus* spp. **A–D, F***C.japonicus* Champion, 1925 **E***C.latus* Pic, 1923 **G***C.amami* sp. n. (paratype).

#### Remarks.

This genus is similar to *Erichia* Reitter, 1895 (= *Jaechobyrrhinus* Pütz, 1991), in general appearance, but differs from the latter in the following characteristics (Pütz 1991): body oblong, gently tapering posteriorly (elongate and subparallel-sided in *Erichia*); pronotum rounded in anterior and posterior corners (pointed in *Erichia*), posterior margin bisinuate (with two rounded extensions in *Erichia*). Most species of this genus have similar external features and intraspecific variation, and cannot be reliably identified without examination of the male genitalia (Pütz 1998).

##### Key to males of the species of the genus *Cephalobyrrhus* of Japan and Taiwan

**Table:** 

1	Phallobase long, 3.3 times as long as parameres; median lobe pointed at apex. Distribution: Japan (Amami-Ôshima)	***C.amami* sp. n.**
–	Phallobase moderate in length, 2.3 times as long as parameres; median lobe obtuse at apex	**2**
2	Phallobase slightly tapering basally; parameres obtuse at apices; median lobe widened in basal 1/3. Distribution: Taiwan	*** C. latus ***
–	Phallobase subparallel-sided; parameres curved interiorly and rather pointed at apices; median lobe straight in sides. Distribution: Japan (Honshu to Kyushu)	*** C. japonicus ***

### Cephalobyrrhus latus

Taxon classificationAnimaliaColeopteraLimnichidae

Pic, 1923

Figs 1A , 2E , 3A–G

Cephalobyrrhus latus : Pic 1923: 4; Champion 1925: 174; Satô 1965: 123; Pütz 1991: 132; 1998: 357.

#### Specimens examined.

1 ex. (SEHU), Alishan Chiayi, 4–5. VII. 1975, H Takizawa leg.; 1 ex. (SEHU), Fenchihu, Nantou, 11–12.VII.1981, H Takizawa leg.; 4 exs. (EUMJ), same loc., 7.VII.1961, S Ueno leg.; 2 exs. (EUMJ), Fushan, Taipei Hsien, 12.IV.1999, Y-Y Lien leg.; 5 exs. (TARI), same loc., 30.III.2012, C-F Lee leg.; 1 ex. (EUMJ), Hsileng, Taoyuan Hsien, 3.V.1981, S Tsuyuki leg.; 1 ex. (EUMJ), Kuanshan, Yakou, Taichung Hsien, 2,600 m, 11.VI.1989, M Satô leg.; 4 exs. (EUMJ), Liyuan (1800–1900 m), Haiduan, Taichung Hsien, 5.VI.2013, K Sonaka leg.; 1 ex. (EUMJ), Mt. Lala, Taoyuan Hsien, 30.IV.1979, S Tsuyuki leg.; 1 ex. (EUMJ), same loc., 2.V.1981, S Tsuyuki leg.; 1 ex. (TARI), Nantou, Tatachia, 6–12.V.2008, C-S Tung leg.; 17 exs. (EUMJ), Nihonmatsu-Hokuko, Byoritsu-ken, 10.IV.1967, T Shirozu leg.; 1 ex. (EUMJ), Oiwake, 24.VI.1961, T Shirozu leg.; 25 exs. (EUMJ), Paolai, Kaosiung Hsien, 11.VI.1989, M Satô leg.; 3 exs. (EUMJ), Sihling-Sicun, Taoyuan Hsien, 19.IV.2007, S-T Hisamatsu leg.; 1 ex. (EUMJ), Sungkang-Meifeng, Nantou Hsien, 19.V.1969, S Hisamatsu leg.; 5 exs. (EUMJ), Tsuifeng, Nantou Hsien, 18.VI.1989, M Satô leg.; 4 exs. (SEHU), Tungpu Chiayi, 14–17.VII.1976, H Takizawa leg.; 4 exs. (EUMJ), Wulai, Taipei Hsien, 4.V.1968, Y Watanabe leg.; 4 exs. (EUMJ), same loc., 17.V.1972, M Sakai leg.

#### Description.

For full description see Pütz (1998). Antennae (Fig. 2E) long, approximate ratio of each antennomere (n = 1) as 2.20 : 1.40 : 2.60 : 1.80 : 1.80 : 1.60 : 1.40 : 1.40 : 1.20 : 1.00 : 1.80. PW/PL 1.57–2.10 (1.79); EL/EW 1.46–1.71 (1.55); EL/PL 3.00–4.29 (3.47); EW/PW 1.17–1.33 (1.24); TL/EW 1.92–2.13 (2.00). Legs reddish brown, but frequently infuscate.

**Figure 3.**
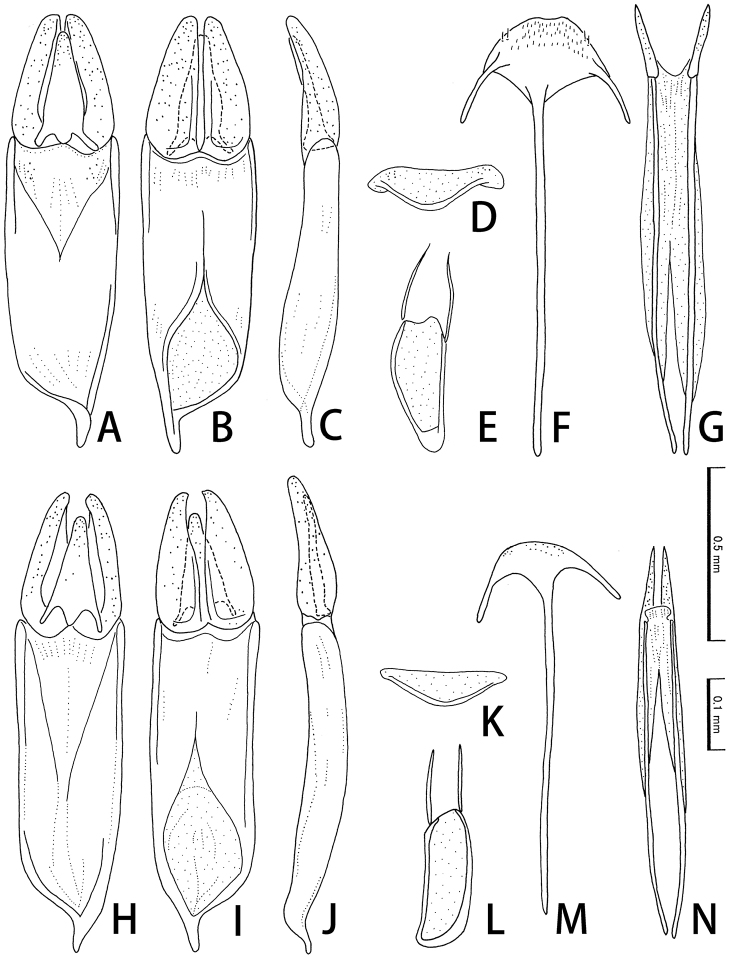
Male (**A–E, H–L**) and female (**F, G, M, N**) genitalia of *Cephalobyrrhus* spp. **A–G***Cephalobyrrhuslatus* Pic, 1923 **H–N***C.japonicus* Champion, 1925 **A–C, H–J** Aedeagus in ventral (**A, H**), dorsal (**B, I**), and lateral (**C, J**) aspects **D, K** sternite VIII **E, L** sternite IX **F, M** spiculum ventral **G, N** ovipositor. Scale bars: 0.1 mm(**A–C, H–J**); 0.5 mm(**D–G, K–N**).

Male. Sternite VIII (Fig. 3D) membranous, transverse. Sternite IX (Fig. 3E) with long and slender lateral struts. Aedeagus 0.6 mm; phallobase slightly tapering basally, with straight basal projection; parameres relatively wide, obtuse at apices, slightly arcuate in lateral sides, 0.42 times as long as phallobase; median lobe widened in basal 1/3, obtuse at apex, 0.88 times as long as parameres.

Female. Spiculum ventral (Fig. 3F) long, as long as ovipositor. Ovipositor (Fig. 3G) well sclerotized, long; coxite gently pointed at apices; approximate ratio of coxite and baculus (n = 1) as 1.0 : 5.0.

Measurements of adults (unsexed; n = 20). TL 2.22–3.70 (3.04) mm; PW 0.88–1.50 (1.22) mm; PL 0.42–0.85 (0.69) mm; EL 1.80–2.85 (2.36) mm; EW 1.05–1.85 (1.52) mm.

#### Distribution.

Taiwan.

#### Remarks.

The male genitalia of the lectotype shown by Pütz (1998) were broken in the basal part.

#### Biological notes.

This species is common in Taiwan, and lives in a natural forest of a mountainous area. Immature stages are unknown.

### Cephalobyrrhus japonicus

Taxon classificationAnimaliaColeopteraLimnichidae

Champion, 1925

Figs 1B , 2A–D, F , 3H–N , 5A–B Japanese name: Oome-hoso-chibidoromushi

Cephalobyrrhus japonicus : Champion 1925: 176; Satô 1966: 62; Pütz 1991: 132; 1998: 352.

#### Specimens examined.

[Honshu] Tokyo Met. 15 exs. (SEHU), Nippara, 24.VI.1969, H Takizawa leg.; 26 exs. (SEHU), Takao-san, 20.VI.1969, H Takizawa leg. Chiba Pref. 3 exs. (EUMJ), Orikisawa, Kimitsu-shi, 12.VI.2013, A Saito leg. Kanagawa Pref. 3 exs. (EUMJ), Miyanoshita, 1.VI.1895; 1 ex. (EUMJ), Yokohama. Yamanashi Pref. 3 exs. (EUMJ), Koganezawa-rindo, 29.VI.1975, S Tsuyuki leg.; Nagano Pref. 1 ex. (EUMJ), Shimajima, 22.VII.1967, M Tomokuni leg. Aichi Pref. 2 exs. (EUMJ), Hongu-san, 15.VI.1991, M Satô leg. Mie Pref. 1 ex. (EUMJ), Hirakura, Misugi-mura, 14.VII.1993, N Kanie leg.; 1 ex. (EUMJ), same loc., 19.VI.1955, Z Naruse leg.; 1 ex. (EUMJ), same loc., 24.VIII.1956, M Satô leg. Gifu Pref. 1 ex. (EUMJ), Suhara, 24.V.1955, K Ohbayashi leg.; 1 ex. (EUMJ), Uchibami-dani, Itadori-mura, 1.VII.1973, Y Hori leg.; 4 exs. (EUMJ), Wara-gawa, 24.VIII.1967, M Satô leg. Tottori Pref. 1 ex. (EUMJ), Daisenji, Daisen, 8.VII.1971, S. Hisamatsu leg. [Shikoku] Ehime Pref. 1 ex. (EUMJ), Fukumi-gawa, 20.VI.1986, S Hisamatsu leg.; 1 ex. (EUMJ), Ishizuchi-san, 16–17.VII.1977, M Tomokuni leg.; 1 ex. (EUMJ), Koguchi, 26.VII.1958, M Miyatake leg.; 1 ex. (EUMJ), Komenono, Matsuyama-shi, 4.VII.1978, Y Seiyama leg.; 2 exs. (EUMJ), same loc., 14.VI.1978, Y Seiyama leg.; 1 ex. (EUMJ), same loc., 4.VII.1974, Y Notsu leg.; 1 ex. (EUMJ), same loc., 13.VI.1976, Y Notsu leg.; 1 ex. (EUMJ), same loc., 23.VI.1977, A Oda leg.; 1 ex. (EUMJ), same loc., 28.VI.1970, M Sakai leg.; 1 ex. (EUMJ), Matsubagawa, 8.VII.1961, M Miyatake leg.; 1 ex. (EUMJ), Myojingamori, 26.VI.1966, S Hisamatsu leg.; 3 exs. (EUMJ), Oda-cho, 10.VII.1983, E Yamamoto leg.; 1 ex. (EUMJ), Odamiyama, 21.VII.1993, M Sakai leg.; 1 ex. (EUMJ), same loc., 27.VI.1972, M Sakai leg.; 1 ex. (EUMJ), same loc., 16.VIII.1972, M Sakai leg.; 2 exs. (EUMJ), same loc., 1–2.VII.1995, N Ohbayashi leg.; 1 ex. (EUMJ), same loc., 18.V.1986, E Yamamoto leg.; 1 ex. (EUMJ), same loc., 19.VII.1993, K Okada leg.; 14 exs. (EUMJ), Ohnogahara, 4.VII.1977, A Oda leg.; 2 exs. (EUMJ), same loc., 11.VII.1965, N Ohbayashi leg.; 3 exs. (EUMJ), same loc., 28.V.1980, A Sakai leg.; 4 exs. (EUMJ), same loc., 6.VII.1979, Y Seiyama leg.; 1 ex. (EUMJ), same loc., 14.VII.1981, M Kotani leg.; 3 exs. (EUMJ), same loc., 14.VII.1981, K Sasagawa leg.; 17 exs. (EUMJ), Omogokei, 26.VI.1951, M Miyatake leg.; 3 exs. (EUMJ), same loc., 15–17.VI.1956, M Miyatake leg.; 6 exs. (EUMJ), same loc., 12–13.VI.1954, S Hisamatsu leg.; 3 exs. (EUMJ), same loc., 19.VI.1955, M Miyatake leg.; 2 exs. (EUMJ), same loc., 26.VI.1955, S Hisamatsu leg.; 6 exs. (EUMJ), same loc., 2.VII.1978, A Oda leg.; 8 exs. (EUMJ), same loc., 29.VI.1963, M Miyatake leg.; 7 exs. (EUMJ), same loc., 21.VII.1979, M Satô leg.; 3 exs. (EUMJ), same loc., 17.VII.1977, A Oda leg.; 1 ex. (EUMJ), same loc., 20.VI.1981, K Sasagawa leg.; 1 ex. (EUMJ), same loc., 14.VII.1981, K Sasagawa leg.; 4 exs. (EUMJ), same loc., 18.VI.1986, T Nagata leg.; 1 ex. (EUMJ), same loc., 12.VI.1959, M Miyatake leg.; 23 exs. (EUMJ), Oonaru, Omogo, 13–14.VI.1998, T. Kan et al. leg.; 2 exs. (EUMJ), Saragamine, 26–27.VI.1959, M Satô leg.; 1 ex. (EUMJ), Shiraitono-taki, 30.VI.1978, I Amano leg.; 3 exs. (EUMJ), Shiratsue, 10–11.VI.1972, M Sakai leg.; 1 ex. (EUMJ), same loc., 19.VI.1966, S Hisamatsu leg.; 1 ex. (EUMJ), Sugitate, Matsuyama-shi, 14.VI.1955, Y Wake leg.; 1 ex. (EUMJ), Wakayama, Kumakogen-cho, 28.VI.2009, K Hashimoto leg. Kagawa Pref. 1 ex. (EUMJ), Daisenzan, Kotonami-cho, 9.VI.2002, Y Kamite leg. Tokushima Pref. 1 ex. (EUMJ), Mimune, Higashi-iya, 2–3.VIII.1969, S Kinoshita leg.; 2 exs. (EUMJ), Takashiro-yama, 17.VII.1988, M Sakai leg.; 1 ex. (EUMJ), same loc., 18.VII.1978, A Oda leg.; 1 ex. (EUMJ), same loc., 18.VII.1978, M Tomokuni leg.; 9 exs. (EUMJ), Tsurugi, 11–12.VII.1976, S Hisamatsu et al. leg. Kochi Pref. 11 exs. (EUMJ), Kage, 11.VI.1971, M Tomokuni leg.; 5 exs. (EUMJ), Kamioriwatari, Yusuhara, 14–15.VI.1997, M Sakai leg.; 5 exs. (EUMJ), Kuroson, 21.VI.1999, M Sakai leg.; 1 ex. (EUMJ), same loc., 15.VII.1953, E Edashige leg.; 3 exs. (EUMJ), Shimooriwatari, Yusuhara, 14–15.VI.1997, N Ohbayashi leg.; 15 exs. (EUMJ), Tebako-yama, 9–11.VI.1960, M Miyatake leg. [Kyushu] Fukuoka Pref. 3 exs. (EUMJ), Hikosan, 7.VII.1957, M. Miyatake leg.; 5 exs. (EUMJ), same loc., 12.VII.2002, J Ogawa leg.; 1 ex. (EUMJ), Ukiha-cho, 18.VII.1956, N Gyotoku leg. Nagasaki Pref. 1 ex. (EUMJ), Sasuna, Kamiagata, 22.VI.2002, T Kurihara leg.; 1 ex. (EUMJ), same loc., 16.VII.2000, J Ogawa leg. Kumamoto Pref. 2 exs. (EUMJ), Hagi, Izumi-mura, 4.V.1988, K Ishida leg.; 1 ex. (EUMJ), same loc., 3.V.1988, K Ishida leg.; 5 exs. (EUMJ), Ichibusa, Mizukami-mura, 8.VI.1967, S Hisamatsu leg.; 4 exs. (EUMJ), same loc., 11.VI.1972, S Hisamatsu leg.; 1 ex. (EUMJ), Nabenodaira, Takamori, 18.VII.1968, S Kinoshita leg. Oita Pref. 2 exs. (EUMJ), Kozubaru, 9–11.VII.1968, S Kinoshita leg.; 2 exs. (EUMJ), Kurodake, Naoiri-gun, 16.VI.1979, S Nagai leg.; 4 exs. (EUMJ), Kyusuikei, Kusu-gun, 16.VI.1979, S Nagai leg. Miyazaki Pref. 1 ex. (EUMJ), Ebino-kogen, Ebino-cho, 14.VII.2002, J Ogawa leg.; 1 ex. (EUMJ), Shiraiwa-yama, Higashi-usuki, 19.VII.1968, S Kinoshita leg.; 27 exs. (EUMJ), Sobo-san, 6.VII.1980, Y Seiyama leg. Kagoshima Pref. 4 exs. (SEHU), Yakushima, 7.VI.1969, K Kushigemati leg.

#### Description.

Adults. For full description see Pütz (1991). Antennae (Fig. 2F) long; approximate ratio of each antennomere (n = 1) as 1.85 : 1.08 : 1.38 : 1.08 : 1.38 : 1.23 : 1.23 : 1.08 : 1.00 : 1.00 : 1.38. PW/PL 1.51–1.90 (1.68); EL/EW 1.43–1.68 (1.56); EL/PL 2.85–3.76 (3.23); EW/PW 1.15–1.30 (1.23); TL/EW 1.94–2.15 (2.04).

Male. Sternite VIII (Fig. 3K) membranous, transverse. Sternite IX (Fig. 3L) with long lateral struts. Aegeagus about 0.6 mm; phallobase subparallel-sided, with straight basal projection; parameres relatively wide, curved interiorly and rather pointed at apices, 0.42 times as long as phallobase; median lobe straight in sides, obtuse at apex, 0.78 times as long as parameres.

Female. Spiculum ventral (Fig. 3M) long, as long as ovipositor. Ovipositor (Fig. 3N) well sclerotized, long; coxite sharply pointed at apices; approximate ratio of coxite and baculus (n = 1) as 1.0 : 3.9.

Measurements of adults (unsexed; n = 20). TL 2.38–4.02 (3.04) mm; PW 0.95–1.65 (1.21) mm; PL 0.50–1.00 (0.72) mm; EL 1.88–3.02 (2.32) mm; EW 1.20–1.95 (1.49) mm.

#### Distribution.

Japan (Honshu, Shikoku, Kyushu, Tsushima, Yakushima).

#### Remarks.

This record is the first of this species from Tsushima.

#### Biological notes.

This species lives in natural forests (particularly in the Japanese beech tree zone, above ca. 1,000 m elevation) near a small stream (Fig. 5A, B). The adults were observed on the surface of rocks and fallen rotten wood during the daytime, and were sometimes attracted to light. Overwintering probably occurs in the larval stage. Immature stages are unknown.

**Figure 4.**
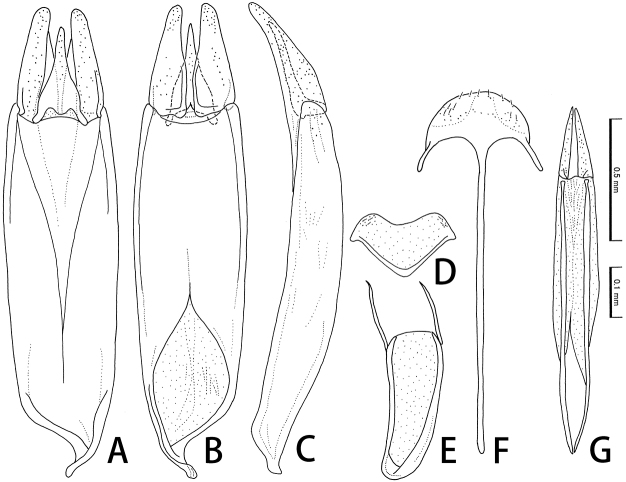
Male (**A–E**) and female (**F, G**) genitalia of *Cephalobyrrhusamami* sp. n. **A–C** Aedeagus in ventral (**A**), dorsal (**B**), and lateral (**C**) aspects **D** sternite VIII **E** sternite IX **F** spiculum ventral **G** ovipositor. Scale bars: 0.1 mm (**A–C**); 0.5 mm (**D–G**).

**Figure 5.**
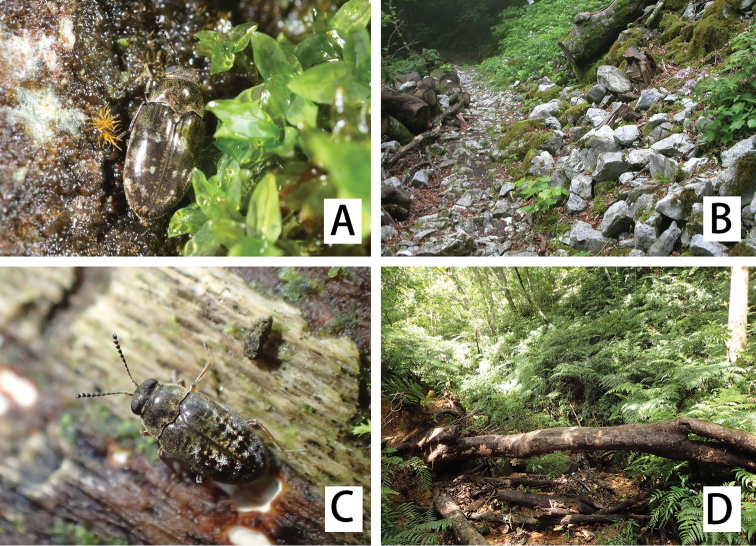
Habitat and habits of *Cephalobyrrhus* spp. in nature. **A***C.japonicus* Champion, 1925 and its habitat **B** Keyaki-daira, Ehime Pref. **C***C.amami* sp. n. and its type locality **D** Akatsuchi-yama.

### Cephalobyrrhus amami sp. n.

Taxon classificationAnimaliaColeopteraLimnichidae

http://zoobank.org/86D9D5E9-A432-4BD0-8578-DF5291C8D435

Figs 1C–D , 2G , 4 , 5C–D Japanese name: Amami-oome-hoso-chibidoromushi

#### Type series.

Holotype (EUMJ): Male, [AM6] Akatsuchi-yama, Uken-son, Amami-Ôshima, Kagoshima Pref., Japan, 28.151853, 129.195539, ca. 107 m, 23.IV.2017, H Yoshitomi leg. **Paratypes** (EUMJ, NMW): 13 males & 2 females, same data as for holotype.

#### Diagnosis.

The new species *C.amami* sp. n. has a short and pointed median lobe and a long phallobase.

#### Description.

Adults. Body oblong, convex dorsally, shiny, densely covered with short golden setae. Coloration of body black; antennomeres I–II, maxillae, labial palpi and legs pale brown, but infuscate in apical part of tarsomeres V.

Head densely punctate, convex anteriorly between antennal insertions. Antennae (Fig. 2G) long, reaching at base of elytra; approximate ratio of each antennomere (n = 1) as 1.78 : 1.22 : 2.00 : 1.11 : 1.33 : 1.11 : 1.00 : 1.11 : 1.11 : 1.00 : 1.22. Pronotum punctate as in head; PW/PL 1.64–1.97 (1.81). Scutellar shield triangular. Elytra oblong, widest at middle, gently arcuate in lateral margins; irregular markings consisting of adpressed setae relatively distinct; EL/EW 1.47–1.56 (1.52); EL/PL 3.06–3.79 (3.41); EW/PW 1.21–1.27 (1.24); TL/EW 1.88–2.01 (1.96).

Male. Sternite VIII (Fig. 4D) membranous, slightly transverse. Sternite IX (Fig. 4E) with long lateral struts. Aedeagus long, 0.9 mm; phallobase long, widest at the middle, then weakly tapering posteriorly and anteriorly, with curved nasal projection; parameres relatively slender, weakly pointed at apices, 0.30 times as long as phallobase; median lobe slender, straightly tapering apically, pointed at apex, 0.89 times as long as parameres.

Female. Spiculum ventral (Fig. 4F) long, a little longer than ovipositor. Ovipositor (Fig. 4G) well sclerotized, long; coxite sharply pointed at apices; approximate ratio of coxite and baculus (n = 1) as 1.0 : 3.8.

Measurements of adults (unsexed; n = 20). TL 3.15–3.65 (3.36) mm; PW 1.30–1.50 (1.38) mm; PL 0.70–0.90 (0.76) mm; EL 2.45–2.75 (2.59) mm; EW 1.60–1.83 (1.71) mm.

#### Distribution.

Amami-Ôshima.

#### Remarks.

This species is clearly distinguished from the other two known species in the region by the short and pointed median lobe and long phallobase.

#### Etymology.

The species is named after the type locality.

#### Biological notes.

This species lives in a natural forest near a small stream (Fig. 5C, D). The type series was collected from the surface of fallen rotten wood during the daytime. Immature stages are unknown.

## Supplementary Material

XML Treatment for Cephalobyrrhus

XML Treatment for Cephalobyrrhus latus

XML Treatment for Cephalobyrrhus japonicus

XML Treatment for Cephalobyrrhus amami
